# Hip arthroscopy via a peripheral compartment first capsular-preserving technique: a step-by-step description

**DOI:** 10.1093/jhps/hnaa061

**Published:** 2020-12-25

**Authors:** Hao-Che Tang, Jason Brockwell, Michael Dienst

**Affiliations:** 1 Chang Gung Memorial Hospital, 222, Maijin Rd., Anle Dist., Keelung City 204, Taiwan; 2 Asia Medical Specialist, 8/F, 29 Queen's Road Central, Central, Hong Kong 999077, Hong Kong; 3 OCM, Orthopädische Chirurgie München, Steinerstraße 6, München 81369, Germany

## Abstract

Hip arthroscopy is a well-recognized procedure for the treatment of several hip pathologies. Different methods of arthroscopic access to the hip have been published. The most popular approach is the central compartment first technique, where the first portal to the central compartment is placed under traction and fluoroscopic control. This technique, however, carries the risk of iatrogenic damage to the cartilage and labrum, especially when adequate distraction cannot be obtained. In addition, secondary exposure of the peripheral compartment frequently requires larger capsulotomies. The current article is to describe an alternative arthroscopic approach to the hip with the peripheral compartment being first accessed. The peripheral compartment first technique offers the advantages of a limited capsular release for peripheral compartment exposure and a reduced risk of iatrogenic cartilage and labrum damage during subsequent central compartment portal placement.

## INTRODUCTION

Arthroscopic access to the hip joint can be commenced either via the central compartment (CC) [1, 2], peripheral compartment (PC) or via an extra-articular (EA) approach [3, 4]. The PC as the primary access for hip arthroscopy was first described by Dorfmann *et al*. in 1988 [5–7] and later modified by Dienst *et al*. [8]. The purpose of this article is to describe the senior author’s current PC first technique of hip arthroscopy. This PC first technique is applicable to the same range of pathologies as other techniques and has certain theoretical advantages: it reduces the chance of iatrogenic injuries to the articular cartilage and acetabular labrum [9] and preserves the capsule. It is particularly applicable in tight joints and coxa profunda [10].

## SURGICAL TECHNIQUE

### Portals

The same skin incisions, with separate capsular incisions, access both the PC and CC. The naming convention is ‘skin incision—compartment’, e.g. the anterior portal (AP) to the central compartment is ‘AP/C’.

**Fig. 1.**
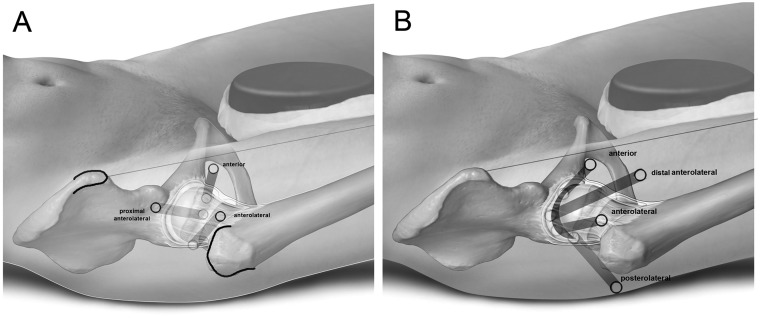
Portals for the peripheral compartment (**A**) and the central compartment (**B**).

Four commonly used portals are arranged in a diamond (Fig. 1). In order of creation, the first portal, at the proximal tip of the diamond, is the proximal anterolateral portal (PAL), used exclusively for visualization in the PC. The second is the AP at the medial point of the diamond, used for viewing and instruments. The third is the anterolateral portal (AL) used mainly for access to the lateral aspect of the PC and to the CC. Finally, the distal anterolateral portal (DAL) allows instruments to reach the anterolateral acetabulum under traction.

### Positioning

Under general anesthesia, the patient is placed in the supine position on a traction table, with a well-padded oversized (15–20 cm diameter) perineal post situated against the perineum. Prior to draping, an unsterile traction test is performed. Manual traction is initially applied to both hips. The non-operative hip is placed in about 20°–30° abduction, neutral rotation and 0° extension. The operative hip is initially placed in neutral rotation, 10° flexion (mild flexion allows a puncture needle to enter the PC while the tension of capsule is maintained) and about 30° abduction. Then the operative hip is brought to about 10° abduction, so the thigh is compressed against the perineal post and a lateral traction force is created. Incremental traction is then applied to the operative hip by turning the traction module, and the degree of distraction is evaluated by fluoroscopy. If there is no distraction and no vacuum sign, rotation of the hip can frequently break the sealing effect and improve the distraction without further traction. After the unsterile traction test, traction on the operative hip is completely released and the patient is draped.

### Access to the PC

**Fig. 2.**
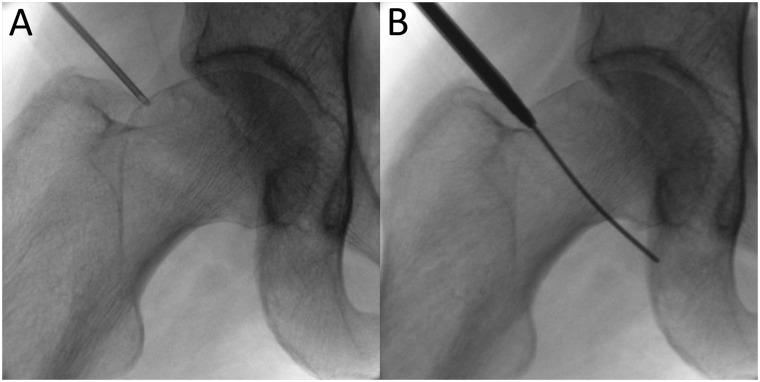
Fluoroscopic imaging of PAL/P placement. The needle is aimed at the head–neck junction and perpendicular to the femoral neck axis. It should penetrate the capsule anterolaterally (**A**). Trocar and shaft are advanced until capsular resistance is felt, and this image is taken to confirm the entry point is sufficiently lateral (**B**).

Most of the work in the PC is performed without traction. The PAL/P, first described by Dienst *et al*. [8], is the key to this technique, and is exclusively used for the arthroscope. It is established first via a skin incision about one-third of the way from the anterior superior iliac spine (ASIS) to the tip of greater trochanter, usually in a soft spot between the medial border of the Gluteus medius and the lateral border of the Tensor fascia lata (Fig. 1A). Under fluoroscopic guidance, a puncture needle is directed perpendicular to the neck axis, about 20° posteriorly and aimed to the head–neck junction. The needle bevel is held downward to facilitate sliding over the anterior surface of the bone as it passes through the capsule. It should penetrate the capsule anterolaterally (at about 1 o’clock in the right hip) and just distal to the head–neck junction, between the physeal scar and the isthmus of the neck (Fig. 2A). This position provides a good view of the whole head–neck junction. If the capsule is penetrated too medially, inspection of the lateral part of the head and neck will be difficult, and the arthroscope may easily slide out of the capsule. A too-distal capsular entry, near the isthmus of the femoral neck, will penetrate the zona orbicularis, which will limit both manoeuverability and visualization. On the other hand, a too-proximal insertion puts the femoral head cartilage at risk.

After penetration of the capsule, 20 ml of saline is injected. A positive ‘reflux test’—where the saline flows back out of the needle secondary to the pressure of the distended capsule—confirms the needle tip is intra-capsular. A nitinol guide wire is inserted through the needle. Intra-capsular needle position can also be confirmed by a firm resistance to the nitinol guide wire from the medial capsule (Fig. 2B).

After placement of the nitinol guide wire, the operative hip is flexed to about 30° and slightly internally rotated to relax the iliofemoral ligament as well as to position the femoral head cartilage in the acetabulum, which reduces the risk of iatrogenic chondral damage when the arthroscope is introduced.

Using a cannulated system, the arthroscopy shaft is introduced until there is a resistance from the capsule. A fluoroscopic image is taken to confirm a correct position and parallel orientation of the shaft to the wire without kinking of the wire (Fig. 2B). The shaft is advanced and a 70° arthroscope is then introduced.

**Fig. 3.**
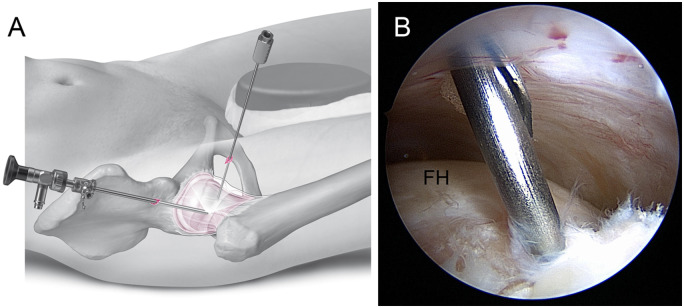
Placement of the AP/P: Illustration (**A**). Arthroscopic inspection of the entry site of needle and nitinol guide wire. The needle should penetrate the capsule at about 3 o’clock and proximal to the zona orbicularis (**B**). FH, femoral head.

A skin incision for the AP/P is made about 5 cm (about three fingers breadths) distal and 30° anterior to the PAL/P, lateral to the sagittal line drawn from the ASIS to the patella. The puncture needle should penetrate the capsule just proximal to the zona orbicularis under arthroscopic control (Fig. 3B). Depending on the anticipated pathology, the perforation of the capsule may be placed more medially (just lateral to the Psoas tendon) for medial synovectomy or more laterally for anterolateral and lateral cam resection.

### Exposure of the PC

Enlargement of the AP/P entry via a capsulotomy is usually not necessary. The shaver is introduced via the AP/P and synovial proliferations are removed, followed by ‘internal thinning’ of the capsule. The process begins with the arthroscope lying at the anterior neck and the shaver working within the anteromedial areas of the PC. Synovial proliferations at the anteromedial perilabral sulcus are removed and the labrum is exposed. When addressing the anteromedial capsule, care must be taken to avoid inadvertent exposure of the Psoas tendon sheath.

The arthroscope is then placed anterior to the femoral head and rotated distally to view the femoral neck and zona orbicularis. Internal release of the zona orbicularis starts anteriorly and advances laterally. The prominent intra-articular thickening of the zona orbicularis is progressively removed from 2 o’clock to 12 o’clock and over to 11 o’clock until the capsule ‘balloons’ from the head–neck junction, providing a good view of the PC. A radiofrequency (RF) device is used to clean up frayed capsular tissue and to release tight sections of capsule.

**Fig. 4.**
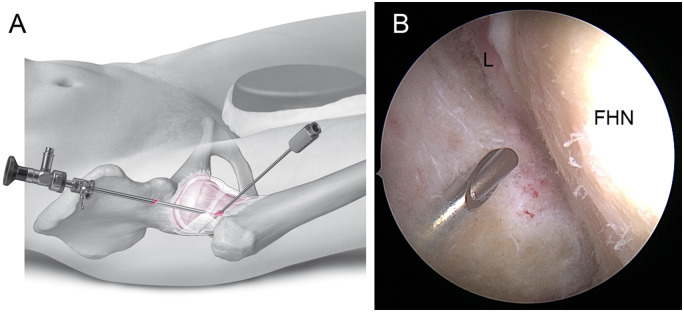
Placement of the AL/P: Illustration (**A**). The needle should penetrate the capsule at about 12 o’clock, 1–2 cm distal to the equator of the femoral head. The switching stick from the AP/P is used to tension the lateral capsule to create space, but is not seen with the arthroscope during puncture of the capsule (**B**). FHN, femoral head–neck junction; L, labrum.

For exposure of the posterolateral area of the PC, the arthroscope at PAL/P is positioned almost vertically for inspection of the lateral and posterolateral capsule. A switching stick is inserted through the AP/P and pushed against the distal-lateral capsule laterally to ‘tent up’ the capsule. The AL/P skin incision is made at about 1 cm proximal and 1 cm anterior to the tip of the greater trochanter, in line with the AP/P. Under arthroscopic control, the puncture needle penetrates the lateral capsule at about 12 o’clock, 1-2 cm distal to the femoral head equator (Fig. 4) and proximal to the ‘tent pole’ switching stick. Because of the strong lateral iliofemoral ligament, a 10–15 mm periportal capsulotomy is made in an anteroposterior direction. Then the shaver or RF device is introduced via the cannulated system.

During exposure of the PC, diagnostic arthroscopy is performed at the same time. The labrum is evaluated with the ‘compression and flip test’ [11], and mechanical conflicts can be assessed by moving the hip into various impingement positions.

Operative arthroscopy of the PC for cam-type femoroacetabular impingement (FAI), synovial diseases, and other pathologic conditions manifesting in the PC, is performed and completed. The hip is moved to different positions in order to allow optimal exposure and access to the different areas of the PC. Usually, treatment starts medially, with the hip in flexion, advanced anteriorly, with the hip in slight flexion, then laterally and posterolaterally, with the hip extended and internally rotated.

### Access to the central compartment

The PC first technique allows portal placement to the CC under arthroscopic control. The hip is brought to neutral extension, and the 70° arthroscope in the PAL/P views the area between the lateral labrum and the lateral femoral head cartilage. An arthroscopy sheath is placed to the lateral perilabral sulcus via the AL/P. The arthroscope is then switched to the AL/P and the area between the labrum and the femoral head cartilage is inspected.

**Fig. 5.**
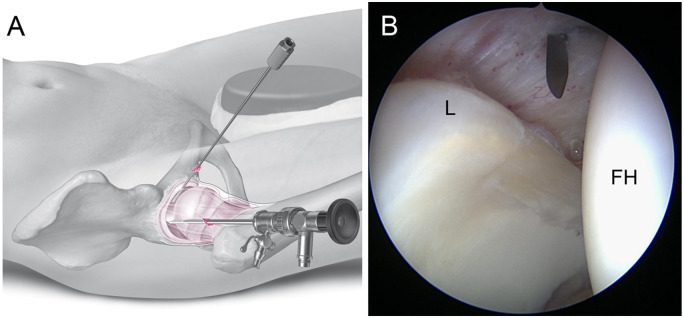
Placement of the AP/C: Illustration (**A**). The needle should penetrate the capsule between femoral head and labrum under arthroscopic control via the AL/P (**B**). FH, femoral head; L labrum.

Under arthroscopic control, the hip is brought to full extension and traction is applied to distract the head from the labrum. The arthroscope is rotated anteriorly to view the anterior capsular triangle between the anterolateral labrum and femoral head. A puncture needle is introduced through the skin incision of AP, entering the CC close to the free edge of the labrum to develop the AP/C (Fig. 5). The periportal capsule is released by about 5 mm medially and laterally, and the arthroscope is switched to the AP/C.

**Fig. 6.**
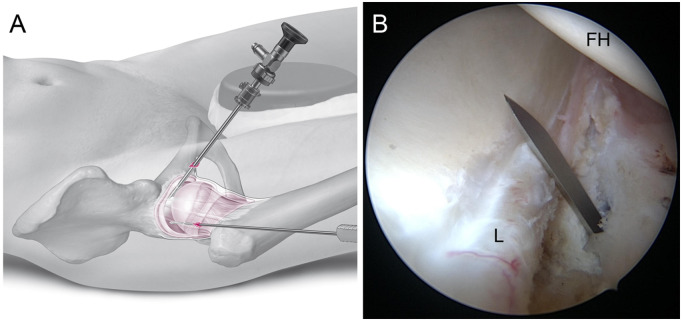
Placement of AL/C: Illustration (**A**). The needle should penetrate the capsule just adjacent to the labrum. The location of penetration is proximal to the AL/P (**B**). FH, femoral head; L, labrum.

Tension of the capsule limits manoeuverability of the instrument in the AL/P and risks femoral head damage, so the arthroscopy sheath is removed from the AL/P, and the puncture needle is reinserted via the AL/P skin incision, entering the CC at about 12 o’clock and distal to the free edge of the lateral labrum (Fig. 6). The capsular perforation is extended about 5 mm anteriorly and posteriorly and an arthroscopy sheath is then advanced into the CC under arthroscopic visualization.

If it is necessary to reach the anterolateral aspect of the acetabulum, the DAL/C is placed when the arthroscope is situated at AL/C for visualization. The skin incision of DAL/C is in line with the PAL, about 8 cm (a palm’s breadth) distally. It allows instruments to reach the anteromedial, anterior and anterolateral labrum, lunate cartilage and acetabular rim. The DAL/C allows drilling of anchor holes with lower risk of perforation into the acetabular cartilage.

For access to the posterior labrum and posteromedial acetabular fossa, including the posteromedial aspects of the teres ligament, an additional posterolateral portal to the CC may be necessary.

### Closure

After diagnostic and therapeutic arthroscopy, traction of the operative hip is released. Instruments are withdrawn, except for a nitinol guidewire placed via the PAL/P to the anterior femoral neck area for optional reentry of the joint. The final contour of the hip is analyzed under fluoroscopy. A needle is introduced along the guidewire and 10 ml of ropivacaine are injected into the joint [12]. The small capsulotomies do not require closure. The skin is closed with interrupted sutures and absorbent dressings are applied.

### Rehabilitation

Immediate physiotherapy and continuous passive and active motion therapy start immediately after the operation to avoid intra-articular adhesions. Range of motion can be increased as tolerated without restriction. Weightbearing is increased to full weightbearing as tolerated over the first 7–10 days. In patients with poor bone quality or who are otherwise considered to be at risk for femoral neck fatigue fracture, partial weightbearing is followed by protected full weightbearing in a 4-point gait for another 2 weeks. After labral repair, partial weightbearing is recommended for 3–4 weeks. After abrasion, microfracture or similar procedures on the cartilage, partial weightbearing is recommended for 5–6 weeks.

## DISCUSSION

To date, the CC first technique is the most popular technique for arthroscopic access to the hip. It is proven to be safe and reproducible [13, 14]. However, the CC first technique has a risk of iatrogenic intra-articular damage. With the hip under traction, one has only fluoroscopy and the feeling of the needle to find its way into the joint. Even with the use of labrum-avoiding techniques [13], occasional iatrogenic damage is sometimes unavoidable. In addition, subsequent exposure of the PC can be more difficult because of periportal and interportal capsulotomies causing loss of iliofemoral ligament tension, resulting in drooping of the capsule onto the head–neck junction, which requires even more extensive capsular work, such as T-capsulotomies [15], or other tricks, for capsular elevation. Moreover, direct approach to the CC can be hindered by acetabular overcoverage, labral ossification and insufficient distraction.

As a consequence, different groups have been searching for alternative approaches. The PC first technique was suggested by Dorfmann and Boyer in 1988 [5–7] and further refined by the senior author of this report [11, 16, 17]. Recently, the EA first technique has been presented [3, 4]. In the EA first technique, the peri-articular space anterior to the joint capsule is accessed before the PC is entered by an anterolateral capsulotomy. This technique offers similar advantages of the PC first technique, but the obligatory larger capsulotomy usually requires capsular repair.

Compared with the CC first technique, the risk of iatrogenic cartilage damage in the PC first technique is low [9]. During the first hip access by PC first technique, the only structure at risk is the anterolateral femoral head cartilage. According to the authors’ experience, a precise fluoroscopy-guided placement to the anterolateral head-neck junction is important to avoid scuffing the cartilage of the femoral head. At the head-neck junction, the needle, trocar and arthroscope are at a relatively safe distance from the femoral head cartilage, particularly if the hip is slightly flexed and internally rotated. Moreover, fluoroscopy allows a precise anterolateral penetration of the capsule at about 1 o’clock (in the right hip) at the level of the head–neck junction.

In addition to the safer first access to the joint, subsequent portal placement to the CC can be visualized from the PC, minimizing the risk of acetabular labrum perforation or femoral head cartilage scuffing. In the ‘early years’ of the PC first technique, the first portal placed to the CC was the AP/C [16]. A precise and safe AP/C placement, however, is limited by the strong anterior iliofemoral ligament. When traction is applied, the space between the anterior head and the anterior capsule is significantly decreased. In addition, the distance between the anterior labrum and femoral head under traction is often smaller than the distance between the lateral labrum and head, especially in acetabular retroversion. Thus, the authors currently prefer placing the AP/C under arthroscopic visualization from the AL/P.

However, access to the CC is technically demanding, no matter which portal is placed first. If the release around the entry site of the AL is inadequate, the pressure from the capsule and soft tissues will increase when traction is applied, and the shaft of the arthroscope situated at AL/P will be pushed toward the femoral head. In this situation, one needs to lever the arthroscope away from the head in order to avoid damage of the femoral head cartilage. In addition, breakage of the vacuum following incremental traction usually causes sudden distal and lateral jumping of the femoral head. The surgeon must anticipate this and place the tip of arthroscope in the perilabral sulcus to prevent damage to the anterolateral femoral head cartilage from the tip of arthroscope.

In the majority of cases, arthroscope and instruments initially placed to the PC need to be redirected during the CC procedure. The reason is that the tension of the capsule and pericapsular muscles will increase after application of traction, and both the arthroscope and instruments in their original PC portal pathways will be pushed toward the femoral head. In addition, redirection of portals with additional capsular perforations close to the free edge of the labrum avoids the need of larger capsulotomies.

In tight joints, acetabular overcoverage, or ossification of the labrum, direct approach to the CC is very difficult or impossible. The PC first technique allows progressive capsular release, resection of the rim or ossifications, and detachment of the labrum from the peripheral side. During or after those steps, traction is applied and access to the CC is feasible in most cases, followed by labral repair if the labrum has been detached. There are, however, few cases where distraction remains inadequate and arthroscopic access to the CC cannot be achieved.

From the authors’ experience, exposure of the PC is more difficult if periportal and interportal capsulotomies are created during the CC first technique. Capsulotomies at the level of the acetabular labrum will result in a decreased tension of the iliofemoral ligament and drooping of the capsule onto the head and neck. As a consequence, visibility within the PC is significantly reduced and a further capsular release is required, frequently leading to vertical extensions of the interportal capsulotomy (T capsulotomy).

A recent study of revisions of arthroscopies performed by a variety of techniques reported 8% incidence of pathological capsular adhesions [18]. In addition to an excellent view of the PC provided by the ‘ballooning technique’, the small capsulotomies heal quickly, which may reduce the risk of arthrofibrosis by early re-establishment of normal synovial fluid circulation and lubrication. However, if the hip is very stiff, more aggressive capsular release or larger capsular incisions without closure may be necessary to allow both adequate exposure and sufficient manoeuverability of instruments. This may be also part of the treatment in order to improve postoperative function. In this condition, the authors prefer a small capsulotomy at the capsular perforation of the AL/P of about 1 cm anteriorly and posteriorly while working in the PC. If exposure and instrumentation of the CC is hindered by a tight capsule, the capsular perforations of both the A/C and AL/C are enlarged parallel to the labrum by about 1 cm on either side. A complete interportal capsulotomy is always avoided.

Criticisms of the described PC first technique include that it is technically demanding, time consuming, with reduced CC visibility due to bleeding after the treatment in the PC, and potential iatrogenic microinstability. There is a learning curve to exposure the PC without creating capsular flaps; however, it is similar to that of capsular repair after large capsulotomies. Poor CC visibility from bleeding after cam resection is not common. This may be related to the authors’ preferred technique of fluid inflow from the CC toward a draining cannula in the PC.

Iatrogenic instability and hip dislocations have been reported as complications after hip arthroscopy with unrepaired large capsulotomies [19–22]. To the authors’ knowledge, frank postoperative instability has not occurred after hip arthroscopies using the PC first technique. However, microinstability may have been overlooked and underdiagnosed. Release of the zona orbicularis may cause subtle instability and impairment of the intra-articular fluid transfer [23]. This needs to be a focus for further observation and research. 
